# Approaches to vaccines against *Orientia tsutsugamushi*

**DOI:** 10.3389/fcimb.2012.00170

**Published:** 2013-01-04

**Authors:** Gustavo Valbuena, David H. Walker

**Affiliations:** Department of Pathology, University of Texas Medical BranchGalveston, TX, USA

**Keywords:** *Orientia tsutsugamushi*, vaccines, immunity, animal models, scrub typhus

## Abstract

Scrub typhus is a severe mite-borne infection caused by *Orientia tsutsugamushi*, an obligately intracellular bacterium closely related to *Rickettsia*. The disease explains a substantial proportion of acute undifferentiated febrile cases that require hospitalization in rural areas of Asia, the North of Australia, and many islands of the Pacific Ocean. Delayed antibiotic treatment is common due to the lack of effective commercially available diagnostic tests and the lack of specificity of the early clinical presentation. The systemic infection of endothelial cells that line the vasculature with *Orientia* can lead to many complications and fatalities. In survivors, immunity does not last long, and is poorly cross-reactive among numerous strains. In addition, chronic infections are established in an unknown number of patients. All those characteristics justify the pursuit of a prophylactic vaccine against *O. tsutsugamushi*; however, despite continuous efforts to develop such a vaccine since World War II, the objective has not been attained. In this review, we discuss the history of vaccine development against *Orientia* to provide a clear picture of the challenges that we continue to face from the perspective of animal models and the immunological challenges posed by an intracellular bacterium that normally triggers a short-lived immune response. We finish with a proposal for development of an effective and safe vaccine for scrub typhus through a new approach with a strong focus on T cell-mediated immunity, empirical testing of the immunogenicity of proteins encoded by conserved genes, and assessment of protection in relevant animal models that truly mimic human scrub typhus.

## Introduction

Scrub typhus, also known as tsutsugamushi disease [from Japanese words meaning disease (tsutsuga) mite (mushi)], is a severe arthropod-borne bacterial infection that is prevalent in a large geographic area, which extends from Afghanistan in the west to Japan, Philippines, and New Guinea in the east, and from the North of Australia in the south to Russia in the north. The disease is transmitted by larval trombiculid mites, known as chiggers (Kitaoka et al., 1974), and is caused by *Orientia tsutsugamushi*, an obligately intracellular bacterium that mainly infects myeloid cells in the inoculation eschar at the beginning of the infection (Paris et al., 2012) and subsequently endothelial cells lining the vasculature once the infection becomes systemic (Moron et al., 2001; Hsu and Chen, 2008). Monocytes and macrophages in all organs are secondary targets too (Moron et al., 2001). *Orientia tsutsugamushi* is a strictly intracellular bacterium that resides free in the cytoplasm of target cells, a characteristic shared by the other genus of the family Rickettsiaceae, *Rickettsia*. *Orientia* first attaches to target cells, possibly using host surface proteoglycans (Ihn et al., 2000; Kim et al., 2004) and bacterial surface proteins such as TSP56 and ScaC (Ha et al., 2011), proteins that can interact with host fibronectin. This interaction appears to facilitate bacterial entry (Lee et al., 2008) via induced phagocytosis (Urakami et al., 1983) mediated by clathrin (Chu et al., 2006), a process that could be triggered through integrin α 5β1. The subsequent down-stream signaling leads to cytoskeletal reorganization (Cho et al., 2010a); orientiae then rapidly escape into the cytoplasm possibly through enzymatic processes that have not been fully characterized.

The disease was first reported in the western scientific literature in 1878 (Palm, 1878) and 1879 (Baelz and Kawakami, 1879). Although the clinical and pathological similarity with other rickettsioses, specifically with Rocky Mountain spotted fever (caused by *Rickettsia rickettsii*), was highlighted as early as 1908 (Asburn and Craig, 1908), the nature of the etiologic agent was obscure. The agent of scrub typhus has been studied since 1906, and was described in detail by Hayashi (1920). Although he believed that a type of *Theileria* (a protozoan) caused scrub typhus, his drawings accurately depict the etiological microorganism as we know it today. In 1924, the agent of scrub typhus was proposed to be a bacterium (Nagayo et al., 1924), and it was identified as a *Rickettsia* by N. Ogata in Japan in 1929 (Sasa, 1967). A clear demonstration was provided by Nagayo et al. in 1930 using intraocular injections in rabbits, guinea pigs, and monkeys. They observed that the organism grows best in endothelial cells of Descemet's membrane (Nagayo et al., 1930). The names *Rickettsia tsutsugamushi* and *R. orientalis* were used (Allen and Spitz, 1945) until 1995 when it was reclassified in a separate genus, *Orientia* (Ohashi et al., 1995; Tamura et al., 1995). Until recently, the sole species of the genus was *O. tsutsugamushi*; however, a second species, *O. chuto*, was discovered in a febrile patient who acquired the infection in the United Arab Emirates (Izzard et al., 2010), and a possible third species may be present in Chile (Balcells et al., 2011).

Scrub typhus presents as a febrile illness with a spectrum of severity ranging from mild to lethal. The less severe form is very difficult to distinguish clinically from murine typhus, which is highly prevalent in the same geographical areas where scrub typhus is present. However, early investigators noticed that murine typhus tends to be a more urban illness while scrub typhus is more of a rural disease (Lewthwaite and Savoor, 1968). Initial tests with differential agglutinating reactions against *Proteus* provided one way to tell them apart (Fletcher et al., 1929), and, subsequently, indirect immunofluorescence assays with specific antigens offered a more definitive method to distinguish them. Immunologically naive patients that acquire scrub typhus, unlike those with murine typhus, frequently develop an eschar (area of necrosis and sometimes ulceration) at the site of inoculation together with regional lymphadenopathy. A maculopapular rash may or may not be present. The outcome is determined by multiple factors but the infecting strain of *O. tsutsugamushi* is believed to be one of the most important ones (Kim et al., 2011), and it seems clear that bacterial proliferation (as opposed to immunopathology) and the time of antibiotic control of the infection are very important predictors of lethality (Sonthayanon et al., 2009). Indeed, delayed treatment leads to complications such as adult respiratory distress syndrome, disseminated intravascular coagulation, acute renal failure, meningitis, meningoencephalitis, and gastrointestinal tract bleeding (Hsu and Chen, 2008; Kim et al., 2011; Yasunaga et al., 2011).

More than one billion people living in those areas of Asia and the Pacific, mentioned above are at risk of acquiring the infection (Watt and Parola, 2003; Watt and Walker, 2006). Where documented, scrub typhus can account for up to 20% of acute undifferentiated febrile hospitalizations in rural areas (Phongmany et al., 2006; Suttinont et al., 2006; Kasper et al., 2012). More than one million cases have been estimated to occur every year; however, this is surely an underestimation because, given the variety of strains and the short-lived humoral immunity, seroepidemiological studies must fail to capture all previous cases (Park et al., 2010). In addition, new areas of endemicity continue to be identified (Chrispal et al., 2010; Zhang et al., 2010). Scrub typhus has also had a dramatic impact on exposed soldiers in the eastern hemisphere since World War II, and is now emerging as an important disease for travelers to the region (Jensenius et al., 2004; Nachega et al., 2007). The development of a prophylactic vaccine to prevent scrub typhus is very important because of at least four other reasons: (1) mortality from this disease can be very high; (2) the clinical presentation, particularly early in the symptomatic period (acute undifferentiated febrile syndrome), is not specific, making clinical diagnosis very difficult; (3) there are no commercially available diagnostic tests that can be used in the rural setting during the acute presentation, making on-site laboratory diagnosis generally impossible (Koh et al., 2010); and (4) in addition to natural resistance to many types of antibiotics, including fluoroquinolones, penicillins, and aminoglycosides, forms of the disease that do not respond well to currently recommended antibiotics (i.e., tetracyclines and chloramphenicol) (Panpanich and Garner, 2002) have been described (Watt et al., 1996; Rosenberg, 1997). Arguments in support of a renewed effort to develop a vaccine to prevent scrub typhus are summarized in Table 1.

**Table 1:** **Arguments in support of the development of a prophylactic vaccine against scrub typhus**.

Scrub typhus is prevalent in a very large geographical area with an ever-increasing population at risk Scrub typhus is responsible for a large proportion of severe undifferentiated fevers in rural areas of Asia and islands of the Pacific Scrub typhus is difficult to diagnose due to the initial non-specific clinical presentation and the lack of sensitive and specific diagnostic tests that can be deployed in rural areas during the acute presentation Mortality can be very high if not treated early There are many strains of the etiologic agent, Orientia tsutsugamushi, and those strains do not stimulate an effective cross-protective immune response Experimental infections of humans show that homologous immunity wanes over time and heterologous immunity is very short-lived *O. tsutsugamushi* is naturally resistant to many actively used antibiotics

## Vaccines against scrub typhus

Widespread awareness of the importance of scrub typhus arose with World War II as large numbers of non-immune troops acquired the disease, sometimes in epidemic proportions, with mortality as high as 37.5% and prolonged convalescence of more than 3 months (Megaw, 1945; Philip, 1948). In addition, DDT, which was extremely effective in killing lice and preventing transmission of epidemic typhus, did not control the mites that transmit scrub typhus. As a consequence, scrub typhus became a priority for prevention and/or therapeutic control. In fact, large-scale production of a vaccine from the processed lungs of cotton rats infected intranasally with *Orientia* was undertaken toward the end of the World War II (Buckland and Dudgeon, 1945). Evidence of protection provided by this vaccine in humans initially came from the analysis of 16 cases that received at least one dose of the vaccine during incubation of naturally-acquired scrub typhus; all but one of these patients had a mild disease (Walker, 1947). However, subsequent analysis of cases of scrub typhus occurring in fully-vaccinated military personnel showed that severe and nearly fatal cases still occurred and that the incidence was not reduced (Card and Walker, 1947). A later human field study in Japan did not show evidence of protection after vaccination with formalin-killed organisms of the Volner strain of *O. tsutsugamushi* (Berge et al., 1949). Improvements to the production method included the use of white rats intravenously inoculated with yolk sac stock of *O. tsutsugamushi* (Smadel et al., 1946). Antigen for vaccination was also produced from fragments of chicken embryos seeded on agar (which presumably produced chicken embryo fibroblasts) and infected with yolk sac-cultivated oriental stocks. A formalin-inactivated immunogen was even produced as late as 2006 from chicken egg (yolk sac)-adapted *O. tsutsugamushi* (Choi et al., 2006). The preparation produced protection in mice infected with the same strains used for vaccination, but there was no protection against other strains.

In 1939, Kawamura et al. infected human volunteers with the Pescadores strain of *O. tsutsugamushi* and showed that when challenged with a very virulent strain, they were solidly protected (Kawamura et al., 1940). Since the Pescadores strain produces a very mild disease, this experiment established some proof-of-concept that an attenuated strain could be an alternative avenue to pursue for vaccine development against *O. tsutsugamushi*. On this topic, it is important to mention that attenuation for mice may not translate directly to attenuation for humans; for instance, Jackson et al. discuss that the egg-adapted Gilliam strain is attenuated for mice, but produced severe infections in human volunteers (Jackson and Smadel, 1951). After the discovery of chloramphenicol, and its clinical effectiveness in scrub typhus (Smadel et al., 1949), Smadel et al., tried a somewhat similar approach by infecting with live *Orientia* followed by antibiotic treatment beginning 1 week after inoculation (Smadel et al., 1951). They observed appropriate protection against a challenge with the same strain (Gilliam). Although this homologous protection lasted for at least 14 months in 11 volunteers who were challenged with the same strain, two did show asymptomatic rickettsemia and one developed clinical illness. On the other hand, when 10 volunteers where challenged a year later with the heterologous Karp strain, they all developed scrub typhus; the disease was immediately treated with chloramphenicol (Smadel et al., 1952a). It is interesting to note that some level of immunity might have been present in the heterologous challenge situation since 4 of 10 immune volunteers did not develop eschars while all non-immune controls did develop eschars (this phenomenon may explain the lack of eschars in many cases in endemic regions where previous infection may have occurred). The eight controls, which were then immune to the Karp strain, were challenged with the heterologous Gilliam strain a month later, and three developed scrub typhus.

A modification of the Smadel-induced infection-treatment method was reported in 1967 (Kekcheyeva, 1967, 1968). In this case, infection with live *O. tsutsugamushi* was treated with tetracycline before vaccination (dubbed chemovaccine or antibiovaccine), a procedure that triggered protection without vaccine-induced mortality in mice. When tested on 21 human volunteers by subcutaneous inoculation, no skin reactions were observed, and only a minority of volunteers developed mild fever or local lymph node tenderness. All volunteers seroconverted; however, no challenge experiments were performed to evaluate protection. Eisenberg and Osterman formulated an alternative to the chemovaccine. It consisted of *O. tsutsugamushi* irradiated with 300,000 rads (cGy). In the BALB/c mouse model, this procedure results in complete protection against a homologous challenge and considerable protection against a heterologous challenge (Eisenberg and Osterman, 1977), although the heterologous protection disappears within 6 months (Eisenberg and Osterman, 1978). This loss was successfully addressed by vaccinating with multiple irradiated strains (Eisenberg and Osterman, 1979). Table 2 summarizes vaccine development efforts for scrub typhus.

**Table 2:** **Vaccines for scrub typhus**.

**Immunogen**	**Characteristics**	**References**
Formalin-fixed homogenized lungs from *Orientia*-infected cotton rats	Not effective in humans	Buckland and Dudgeon, 1945; Card and Walker, 1947
Formalin-killed *O. tsutsugamushi*	Not effective in humans	Berge et al., 1949
Formalin-killed *O. tsutsugamushi*	Limited homologous protection in mice	Bailey et al., 1948; Rights and Smadel, 1948; Choi et al., 2006
Live strain of *O. tsutsugamushi* with low virulence	Solid protection in humans	Kawamura et al., 1940
Inoculation of live virulent strain followed by antibiotic treatment	Protection against homologous strain only and evidence of persistent infection with immunizing strain in humans	Smadel et al., 1951; Kekcheyeva, 1967, 1968
Live irradiated *O. tsutsugamushi*	Complete protection against homologous strains and poor protection against heterologous strains in mice	Eisenberg and Osterman, 1977, 1978, 1979
Recombinant 56-kDa protein	Protection against homologous strain in mice	Seong et al., 1997a,b
Recombinant fragment of 56-kDa protein	Only partial protection against homologous strain in NHPs	Chattopadhyay et al., 2005
DNA encoding 56-kDa protein	Partial protection against homologous strain in mice	Ni et al., 2005
Recombinant fusion of 56-kDa and 47-kDa proteins	Partial protection against homologous strain in mice	Yu et al., 2005

## Strain diversity

The issue of *Orientia* diversity was first suggested from the epidemiological data demonstrating large differences in severity of outbreaks depending on geographical location (mortality rates ranging from 0.6% to 35% in allied forces during WWII) (Philip, 1948), lack of serological cross-reactivity in some confirmed human cases (Philip, 1948), and suggestions of reinfection in humans (Romeo, 1946; Kuwata, 1952) and non-human primates (NHPs) (Kouwenaar and Esseveld, 1949). In the report by Kuwata et al., they included a bibliography in Japanese with many cases of reinfection reported since 1911. Other studies characterized the antigenic heterogeneity of *O. tsutsugamushi* through the use of immune rabbit sera against panels of distinct isolates of the bacterium by direct immunofluorescence (Shirai et al., 1979b) or through a plaque reduction assay (Oaks et al., 1980). Subsequently, after the monoclonal antibody technology was developed, it became possible to prove that there are antigens private to each strain (Eisemann and Osterman, 1985; Park et al., 1993), and phylogenetic analysis of the gene encoding an important immunodominant antigen, the 56 kDa protein (or TSP56), showed different levels of homology among many strains (Ohashi et al., 1996; Enatsu et al., 1999; Ruang-Areerate et al., 2011). A recent analysis of the sequences of genes encoding surface antigens (*sca*) from four strains of *O. tsutsugamushi* showed that there are variable levels of conservation and even absence of some genes in some strains (Ha et al., 2012). The predominance of sequence variation at the level of surface genes had already been noted when the two fully sequenced *O. tsutsugamushi* genomes (strains Boryong and Ikeda) were analyzed (Nakayama et al., 2008, 2010). In fact, the genomic evidence suggests that transposable elements have continued their amplification and decay after divergence of the two sequenced strains. This may explain the extreme degree of genome shuffling that is evident when the two strains are compared.

## Naturally acquired immunity is suboptimal

In 1950, the lack of solid immunity in humans was confirmed through inoculation of volunteers. In this study, 11 of 16 volunteers who had scrub typhus (11–25 months earlier) developed scrub typhus of similar severity as controls after subcutaneous inoculation of *O. tsutsugamushi*, Gilliam strain (Smadel et al., 1950). The fact that none of eight volunteers who had scrub typhus 1–2 months earlier developed the disease again upon rechallenge suggested that immunity is established but that it wanes rapidly. Most of the original infections were believed to have been caused by strains of *O. tsutsugamushi* different from Gilliam (therefore, this was likely a heterologous challenge). In one volunteer from the same study, it was known that the first infection was caused by the same strain of *O. tsutsugamushi* used for the challenge more than 3 years later; this person developed no other symptoms besides localized erythema at the site of inoculation. Furthermore, in another study, a homologous challenge was performed on one individual three and a half years after the first infection, and protection was complete (Smadel et al., 1952a). The picture that emerged from these studies was that, in humans, heterologous immunity is very short-lived while homologous immunity may be long-lasting. This picture is also supported by experiments with NHPs. MacMillan et al., showed that cynomolgus monkeys are susceptible to the homologous strain of *O. tsutsugamushi* when re-infected 5–6 years after the first infection, but not when rechallenged 8 months after the first infection (MacMillan et al., 1985). In contrast to these studies with humans and NHPs, some early studies showed that heterologous immunity in gerbils is strong even after 6–9 months following initial infection and antibiotic treatment with para-aminobenzoic acid (Zarafonetis et al., 1946). Similar findings were reported for guinea pigs (Topping, 1945a) including, in one study, challenges performed nearly 2 years after the initial infection (Kouwenaar and Esseveld, 1949). It is probable that gerbils and guinea pigs are particularly resistant to infection with *O. tsutsugamushi*; indeed, that was the experience of early investigators (Lewthwaite and Savoor, 1968).

## Homologous vs. heterologous immunity

The disparity between protection against heterologous oriental strains stimulated by infection followed by recovery, albeit short-lived, and the lack of heterologous protection afforded by vaccines (Rights and Smadel, 1948) or immune serum (antibodies) was evident to early investigators, but the possible implications could not have been forecasted without a better understanding of the mechanisms of immunity, particularly the role of cellular immunity, which was only first investigated for scrub typhus in 1976 (Shirai et al., 1976). In the study by Rights and Smadel, formalin-killed vaccines prepared from rat lungs and spleens stimulated complete protection against a lethal intraperitoneal challenge with the homologous strain and variable protection against seven other strains with a range from significant protection to absolutely no cross-protection. It should be noted, however, that even mice challenged with the homologous strain developed illness (although they were protected from dying). It is also notable that subcutaneous immunization with formalin-inactivated organisms did not provide protection even against the homologous strain; vaccination and challenge had to be done through the same route, intraperitoneally (Bailey et al., 1948). This is in contrast to live *O. tsutsugamushi*, which provides homologous protection when used for priming via the subcutaneous route (Shishido et al., 1958, 1959). Another study showed that, mice are susceptible to a heterologous challenge during the course of an acute oriental infection (Kuwata, 1952); after day 8 of the acute infection, these mice did not succumb to the heterologous *O. tsutsugamushi*, but the heterologous *O. tsutsugamushi* organisms could be isolated from these mice; therefore, superinfection can occur. This finding appears to be relevant to humans because there is evidence that humans can harbor multiple strains of *O. tsutsugamushi* during a single clinical episode (Elisberg et al., 1967). Unfortunately, there is very little information about the nature of the anti-oriential immune response in individuals from endemic areas that may frequently be exposed to multiple strains.

## Persistence of *O. tsutsugamushi*

For mice, the phenomenon of bacterial persistence was first reported as a Pers. communication from Fox and Peterson to Zarafonetis (Zarafonetis et al., 1946). These authors questioned whether the chronic infection stimulates an “interference phenomenon” (i.e., activated innate immunity) that could explain the initial heightened immunity. Kowenaar, et al. mention in one of their studies that *O. tsutsugamushi* could be found in the urine of humans and guinea pigs even after the end of the symptomatic period (Kouwenaar and Esseveld, 1949). They were able to recover these bacteria from various tissues and fluids of guinea pigs up to 635 days post-infection. Fox demonstrated persistence in mice inoculated i.p., with the Karp strain of *O. tsutsugamushi* (treated with toluidine blue to prevent their death) by inoculating brain or liver (of animals sacrificed up to 62 days later) and blood or kidney suspensions (of animals sacrificed up to 320 days later) into naive mice (Fox, 1948). Also, live *O. tsutsugamushi* was found in most organs of animals treated with methylene blue up to 496 days later. Similarly, in cotton rats, persistence was identified up to 3 months after inoculation.

In a later study in 1964, Kundin et al. tested various stress methods (including irradiation and corticosteroids) to elicit recrudescence from mice infected 1 year earlier with *O. tsutsugamushi* via the subcutaneous route (Kundin et al., 1964). Although they were able to recover *O. tsutsugamushi* from 20 to 25 stressed mice, they accomplished the same (13 of 14 mice) from control unstressed mice. A subsequent study used cyclophosphamide to reactivate the *Orientia* 19 months after the initial infection (Shirai et al., 1979a). In humans, Smadel et al. reported the isolation of *O. tsutsugamushi* from an enlarged axillary lymph node of a person who had recovered clinically from scrub typhus 15 months earlier. The organism was recovered from mice inoculated intraperitoneally with the supernatant of a homogenate of the lymph node. Similar attempts in 11 other volunteers that had recovered from scrub typhus were unsuccessful (Smadel et al., 1950, 1952b). Interestingly, the positive lymph node was very fibrotic with almost complete absence of lymphoid follicles and with prominent thick-walled blood vessels. This volunteer was also challenged with the Gilliam strain of *O. tsutsugamushi* immediately after the lymph node extraction and also 18 months later; on both occasions, no signs of illness developed. In NHPs, *O. tsutsugamushi* can also be recovered from draining and non-draining lymph nodes of otherwise healthy convalescent animals (Walker et al., 1973). In addition, clinically healthy NHPs that had recovered from scrub typhus were not only susceptible to a heterologous challenge, but also showed evidence of recrudescence of the original strain used for the initial infection (Robinson et al., 1981). This finding is very interesting because there is very suggestive evidence of infection of humans with more than one strain of *O. tsutsugamushi* (Elisberg et al., 1967, 1968; Sonthayanon et al., 2010), which could be explained by either recrudescence of an original infection after reinfection with a heterologous strain or by simultaneous infection with different strains. In turn, this latter possibility could be explained by bites from different chiggers, each one infected with only one strain or, alternatively, by the bite of individual chiggers infected with multiple strains. There is indeed evidence that individual chiggers can be infected with multiple strains (Shirai et al., 1982a,b). Most recently, Chung et al. were able to isolate *O. tsutsugamushi* from the blood of six convalescent patients 1–18 months after the initial diagnosis and treatment with azithromycin, doxycycline, or a combination of antibiotics (Chung et al., 2012). All these data strongly suggest that scrub typhus is a chronic infection in an unknown proportion of patients. An additional and very important implication of the simultaneous infection of hosts and chiggers by different strains of *O. tsutsugamushi* is that lateral gene transfer becomes a possibility that could drive the evolution of *Orientia* and the further diversification of strains. This possibility is supported by recent genomic analyses of multiple strains (Duong et al., 2010; Sonthayanon et al., 2010).

The underlying mechanisms of persistence are not known. There clearly are pathogen factors, but host factors related to the immune response must also be in play. In this regard, it has been suggested that the slow development of T cell effector responses may be causally related to the establishment of chronic infections (Miller et al., 2008).

## Effect of the vector

Although the native people of endemic areas were keenly aware of the transmission of scrub typhus by chiggers (Blake et al., 1945), the concept of transmission by mites was first established in the scientific literature by Tanaka in 1899 based on clinical observations and intimate familiarity with the disease as he was a physician practicing in an endemic area of Japan (Sasa, 1967). Only the larval stage of *Leptotrombidium* mites (chiggers) parasitizes vertebrate hosts by feeding on lymph and tissue fluids of the dermal layer (but not blood) for a period of 2–4 days (Philip, 1948). Infected mites maintain the infection through transtadial and transovarial transmission (Phasomkusolsil et al., 2009). In a large proportion of immunologically naive patients, an eschar develops at the bite site. It is very likely that chiggers, just like ticks (Francischetti et al., 2009), have immunomodulatory factors in their saliva that could affect the outcome of the disease; however, this remains an unexplored area. Clues of those effects could be obtained from the study of eschars from human cases. Indeed, a recent study of 11 eschars from patients with confirmed scrub typhus confirmed the classic histological finding of epidermal necrosis and ulceration accompanied by significant neutrophilic infiltration adjacent to the area of necrosis and mononuclear cell infiltrates in the dermis (Lee et al., 2009; Paris et al., 2012). Those infiltrates consisted of T cells, monocytes, and dermal dendritic cells. In fact, monocytes, dermal dendritic cells, and Langerhan's cells were found to be infected with *O. tsutsugamushi* as judged by confocal microscopic analysis. In contrast, infection of endothelial cells was extremely rare in the eschars.

Studies of mites are complicated by their size and the difficulty of breeding them given that the nymph and adult stages do not feed on blood. The first successful breeding was reported in 1951 using mosquito eggs as food as for the late developmental stages of these mites (Sasa, 1967). Subsequently, the experimental transmission of *O. tsutsugamushi* to humans (Shirai et al., 1982b) and ICR mice (Shirai et al., 1982a) using chiggers was reported. Notably, chiggers from the same colonies that produced classic scrub typhus in humans produced minimal morbidity in mice and NHPs. Most recently, it was shown that even one chigger was sufficient to accomplish the infection of ICR mice (Lurchachaiwong et al., 2012). One species of chiggers transmitted a strain of *O. tsutsugamushi* with near complete lethality while two other different mite species transmitted a strain that caused death of about half of the exposed mice. However, it is not possible to determine whether the differential mortality is due to differences in the vector since the strains of *O. tsutsugamushi* were not the same in different chigger lines.

## Animal models

The earliest experiments with both mite bite and percutaneous infections were performed by Hayashi using NHPs and guinea pigs (Hayashi, 1920). Subsequently, the majority of published studies have used mice infected with *O. tsutsugamushi* via the intraperitoneal route, which produces an intense localized infection of the peritoneum and all serosal surfaces within it (Ewing et al., 1978), but not a significant disseminated endothelial infection, which is the hallmark of human scrub typhus (Moron et al., 2001). In fact, infection of mesothelial cells, which line serosal surfaces in the peritoneum, produces a cycle of infection where membrane-enclosed orientiae bud from infected cells and these host membrane-bound bacteria enter, via phagocytosis, other mesothelial cells (Ewing et al., 1978). Whether this same cycle is operational in the true target cells of *O. tsutsugamushi* has not been investigated.

Not unexpectedly, different mouse strains have different degrees of susceptibility to oriental infection (except for the Karp and Kato strains that are equally lethal for all mouse strains) via the i.p. route (Groves and Osterman, 1978) with C3H mice being the most susceptible (as determined with the Gilliam strain). That susceptibility appears to be linked to a single locus (*Ric*) *on* chromosome 5 (Groves et al., 1980) where the gene for early T lymphocyte activation 1 (*Eta-1*, better known as osteopontin) is located (Patarca et al., 1989). Indeed, different alleles of this gene are present in resistant and susceptible mouse strains; susceptible mice show a very delayed increase in this gene's product as compared to resistant mice, which respond within 1 day.

In contrast to macrophages from BALB/c mice, those of C3H mice migrate to the infected peritoneum with delayed kinetics, do not become activated, and are much more susceptible to oriental infection once in the peritoneum (Nacy and Groves, 1981). Since C3H macrophages can be activated *in vitro* to kill *O. tsutsugamushi* in a similar fashion as macrophages from BALB/c mice, the problem appears to be one of regulation of macrophage function. When congenic C3H strains that differ only in the *Ric* locus were compared, the strain with the locus derived from resistant mice (C3H/RV, which is also known to have a better interferon response) was resistant to a lethal challenge with Gilliam strain (Jerrells and Osterman, 1981), and the main difference was that there was a large influx of neutrophils on days 5 and 10 in the susceptible mice, but not in the resistant mice, which suggests that neutrophils could be associated with ineffective immunity. In addition, all infiltrating cells (particularly macrophages and lymphocytes) supported oriental growth while very few of the peritoneal infiltrating cells of resistant mice were infected. It is very interesting to note that Gilliam strain-resistant C3H/RV mice responded to i.p., Karp strain infection with a large influx of neutrophils. In contrast with the differences observed with i.p inoculation, C3H/RV and C3H/He were both resistant to an intravenous inoculum of Gilliam strain that is consistently lethal when given i.p., to C3H/He mice (Jerrells and Osterman, 1982a). These mice developed strong immunity. With a 10-fold higher dose, however, C3H/He mice died after intravenous inoculation of Gilliam strain but not C3H/RV mice. When macrophages were depleted with silica or carrageenan (i.v.), C3H/He mice became susceptible to lethality with the lower i.v. inoculum while C3H/RV mice remained resistant. Thus, monocytes and macrophages appear to play a complex role in immunity against *Orientia*; they are both targets of oriental infection as well as potential effectors of bacterial killing.

As for other routes of experimental infection, intracerebral inoculation produces a localized infection in the brain while intramuscular or subcutaneous infections result in systemic infections, but this effect depends on the virulence of the *Orientia* strain used (Kundin et al., 1964). Importantly, the subcutaneous route causes a chronic infection with rickettsemia detectable for at least 52 weeks after the initial infection (Groves and Kelly, 1989). This phenomenon is associated with maintenance of high levels of specific antibodies.

Rhesus macaques have also been used to study scrub typhus (Morris, 1965). Although it appears that most macaques are resistant to infection with *O. tsutsugamushi*, this resistance is probably due to previous infection as indicated by a high seroprevalence (Heisey et al., 1981). For this reason, Walker et al. developed a model with silvered leaf-monkeys given that they are not likely to be exposed to chiggers in nature since they are arboreal. When infected subcutaneously, these NHPs developed a disease resembling human scrub typhus including formation of an eschar at the inoculation site, draining node lymphadenopathy, fever with a peak in the third week post-inoculation, low mortality (2 of 14 NHPs), seroconversion in surviving animals, and persistence of *O. tsutsugamushi* in lymph nodes at 2 months after clinical recovery in 11 of 12 monkeys and 5 months later in 1 of 2 monkeys (Walker et al., 1973). The duration of fever was longer with the Gilliam strain than Karp or Kato strains. Subsequent studies with this model showed that specific antibodies were not detectable 14 months after the initial challenge in 17 of 18 monkeys and that they were no longer protected when challenged with homologous or heterologous strains of *O. tsutsugamushi*, although the disease was less severe than that of naive controls (Robinson et al., 1981). Subsequently, when seronegative cynomolgus macaques were studied, it was found that they also constituted an excellent model that faithfully mimicked human scrub typhus (MacMillan et al., 1985), including the histopathological characteristics of the inoculation eschar (Walsh et al., 2007).

## The role of antibodies in immunity against *orientia*

Early experiments suggested that antibodies can be protective if present early in the disease course as demonstrated through passive transfer of serum from immune rabbits to mice infected with a lethal intraperitoneal inoculum of *O. tsutsugamushi*, Karp strain (Topping, 1945b). Some of the mice were completely protected, and death was delayed in the rest of the animals when the immune serum was provided as late as 168 h post-infection. Optimism about the use of serum therapy in humans was based on the thought that the inoculation eschar would provide an early sign of the disease to trigger this therapy at a point in the course when, as judged from experiments performed with Rocky Mountain spotted fever in humans, it is most effective. On the other hand, early experiments with the complement-fixation test showed that when strain-specific antisera (from guinea pigs and humans) were titrated against homologous or heterologous strains of *O. tsutsugamushi*, the end-point titers against the homologous strain were always considerably higher than the end-point titers against the heterologous trains (Bengston, 1945; Bailey et al., 1948). Similar results were obtained when neutralization was used as the readout of the experiment (Bennett et al., 1949; Hanson, 1983). In addition, animal experiments with immune sera against different strains showed that neutralization is complete for the homologous strain but does not provide protection against challenges with heterologous strains (Bell et al., 1946). One of the implications of those studies is that diagnostic serological tests are much more sensitive for the detection of antibodies against the homologous strain. The other implication is that heterologous antibody-mediated cross-immunity may not be as potent as homologous immunity. Moreover, anecdotal evidence from human cases indicates that convalescent serum did not impact the progression of the disease (Philip, 1948). Similarly, in guinea pigs, immune serum (from either humans or guinea pigs) did not yield a favorable outcome (Kouwenaar and Esseveld, 1949). An additional issue with humoral immunity in humans is that specific antibodies, as measured by indirect fluorescence assay (IFA) in one study, become undetectable in 40 months (Saunders et al., 1980). The decrease in specific antibody titers against *O. tsutsugamushi* was also recently confirmed in humans (Kim et al., 2010). In NHPs, antibodies are lost within 1 year (MacMillan et al., 1985). These findings are very important not only in terms of protective immunity, but also in terms of the outcomes of seroepidemiological studies that may, in consequence, underestimate the actual prevalence of scrub typhus. Finally, although the antibody response transitions from IgM early after an acute infection of naive individuals (both humans and experimental animals) to IgG, systematic studies of immunoglobulin isotypes and the influence of their effector functions (e.g., complement fixation and antibody-dependent cytotoxicity) on disease outcomes have not been undertaken.

## The role of T cells in immunity against *orientia*

The observation of delayed hypersensitivity after experimental immunization suggests a role for cell-mediated immunity (Jerrells and Osterman, 1982a). One of the first suggestions of the importance of cell-mediated immunity came from studies with the Gilliam strain of *O. tsutsugamushi*, which is less virulent than the Karp strain in BALB/c mice infected by intraperitoneal infection (Catanzaro et al., 1976). In this model, after mice recover from the initial infection with Gilliam strain, they do not have antibodies that cross-react with Karp strain; however, they resist a challenge with the Karp strain (Shirai et al., 1976). Furthermore, transfer of anti-Gilliam serum did not confer protection against a lethal challenge with Karp strain; however, passive transfer of splenocytes (depleted of plastic adherent cells, mainly macrophages) from Gilliam-immune mice to naive mice produced complete resistance against a lethal challenge with Karp strain when the splenocytes were derived from mice infected with Gilliam strain 2–3 weeks earlier and 80% protection when the splenocytes came from mice infected with Gilliam strain 42 or 63 days earlier. The protective effect was abrogated when the cells to be transferred were depleted of T cells. In a subsequent study by the same group, they were able to use the Karp strain for immunization via the i.p., route without causing lethality in mice if the bacteria were gamma-irradiated with a dose that keeps the bacteria alive (as demonstrated by their ability to enter cells *in vitro*) but prevents their replication (Eisenberg and Osterman, 1977; Eisenberg et al., 1980). They reported complete homologous protection and significant heterologous protection against lethal challenges, and, as in the earlier study, the protection was cell-mediated. Interestingly, splenocytes from animals immunized with formalin-inactivated *O. tsutsugamushi* did not confer any protection against either the homologous or heterologous strains after adoptive transfer into naive animals, suggesting that T cell-mediated immunity is not developed with this type of immunization. These results seem to explain the results of early studies and trials with formalin-inactivated *O. tsutsugamushi*.

NHPs also demonstrate antigen-specific proliferation of lymphocytes from peripheral blood against both homologous and heterologous antigens (MacMillan et al., 1985); however, unlike mice, the response declined with time. From the perspective of current knowledge in immunology, this could be due not to loss of responding cells but to migration of memory T cells to relevant organs, which, in consequence, would not be detectable in peripheral blood. From human cases, we know that during the acute phase of scrub typhus (on admission to the hospital) there is elevation of several cytokines such as IFN-γ, IL-18, IL-12(p40), TNF-α, T cell-targeting chemokines (CXCL9 and CXCL10), and of granzymes A and B, which are indicators of NK cell or CD8+ T cell activation, or both (Iwasaki et al., 1997; Chierakul et al., 2004; de Fost et al., 2005). Recent studies of patients with scrub typhus have shown elevation of other cytokines besides those just mentioned including IL-1β, IL-10 (Kramme et al., 2009), and IL-6 (Chung et al., 2008). Also, in C3H/HeN mice infected i.p., with Gilliam strain, these and other cytokines and chemokines (RANTES, Mip1-a, Mip-1b, Mip-2, MCP-1) were identified in peritoneal lavages with peaks that coincided with the degree of leukocyte infiltration (Koh et al., 2004). The levels of these cytokines were higher in susceptible C3H mice than in resistant BALB/c mice (Yun et al., 2005). It is likely that intracellular sensing of orientiae in target cells contributes to cytokine secretion. In this regard, it was recently shown that in *O. tsutsugamushi* infection of endothelial cells the intracellular innate receptor NOD1 leads to production of IL-32, which in turn regulates the production of other cytokines and adhesion molecules (Cho et al., 2010b). The significance of the above findings related to cytokines and chemokines depends on the kinetics and interactions of the various components within the context of the entire system *in vivo*, and this systems approach has not yet been attempted.

The information presented above supports the concept that the anti-oriential humoral immune response is very poorly cross-reactive or not cross-reactive at all for different strains of *O. tsutsugamushi*, while the T cell response is significantly cross-reactive. Jerrells and Osterman provided further supportive evidence in 1983 when they demonstrated that lymphocytes from immunized animals proliferate in response to both homologous and heterologous oriental antigens (Jerrells and Osterman, 1983). The responding lymphocytes were T cells based on lack of adherence to nylon wool, response to concanavalin A, and lack of response after depletion with anti-Thy1.2 serum. A subsequent study showed that these T cells produced cytokines, including interferon (Jerrells et al., 1983), more specifically, IFN-γ (Palmer et al., 1984a,b), which, based on the timing and magnitude of its peak level, was one of the first cytokines to be considered very important (Palmer et al., 1984a). Similarly, NHPs' T cells also respond to oriential infection with production of IFN-γ (MacMillan et al., 1985). The *in vivo* relevance was further supported by the protection provided with immune IFN-γ-producing T cells transferred into naive mice (Kodama et al., 1987). Despite the apparent importance of T cell-mediated immunity in the anti-oriential response, it is interesting to note that patients with HIV infection do not develop a more severe form of scrub typhus (Kantipong et al., 1996). In fact, scrub typhus results in a decrease in HIV replication as determined by a comparison of viral loads in HIV-positive patients with scrub typhus vs. HIV-positive patients with other infections (Watt et al., 2000).

Of course, a physiological immune response against an obligately intracellular microbe includes a combination of antibodies and cell-mediated immunity that work together. The importance of this synergistic interaction is suggested by the comparison of the level of protection stimulated by vaccination with irradiated bacteria vs. that induced by live bacteria. Mice immunized with irradiated bacteria produced very little antibody and, although completely protected, had orientemia after challenge. On the other hand, mice immunized subcutaneously with live non-irradiated bacteria had much higher level of antibodies and did not manifest orientemia after a homologous challenge (Jerrells et al., 1983). Also, it is now clear that effective antibody responses are T cell-dependent; this was first demonstrated in studies with athymic mice which were protected by transfer of immune T cells, and only when this transfer was performed did mice develop a humoral immune response (Kobayashi et al., 1985). Table 3 summarizes the characteristics of the natural adaptive immune response against *O. tsutsugamushi*.

**Table 3:** **Adaptive immune response against *O. tsutsugamushi***.

**Species**	**Study findings**	**References**
Humans	Homologous immunity lasts up to 3.5 years but heterologous immunity lasts only a few months	Smadel et al., 1950, 1952a,b
	Humans can be infected by multiple strains simultaneously	Elisberg et al., 1967, 1968; Sonthayanon et al., 2010
	Oriential infections in humans can be chronic	Kouwenaar and Esseveld, 1949; Smadel et al., 1950, 1952a,b Chung et al., 2012
	Humoral immunity, even against the homologous strain, is short lived	Saunders et al., 1980; Kim et al., 2010
Non-human primates	Homologous immunity is non-existent after 5 years	MacMillan et al., 1985
	Oriential infections in NHPs can be chronic	Walker et al., 1973
	A heterologous oriential infection can induce recrudescence of a prior infection	Robinson et al., 1981
	Humoral and cellular immunity are short lived	MacMillan et al., 1985
Rodents	Heterologous protection is present 9 months after infection in gerbils	Zarafonetis et al., 1946
	Heterologous protection is present 2 years after infection in guinea pigs	Topping, 1945a; Kouwenaar and Esseveld, 1949
	Mice can be infected by a different oriential strain during the course of an acute oriential infection	Kuwata, 1952
	Oriential infections in mice can be chronic	Zarafonetis et al., 1946; Fox, 1948; Kundin et al., 1964; Shirai et al., 1979a
	Antibodies provide homologous protection but are poorly cross-protective	Bengston, 1945; Topping, 1945b; Bell et al., 1946; Bailey et al., 1948; Bennett et al., 1949; Hanson, 1983
	T cells provide homologous protection and significant heterologous protection. IFN-γ- is a particularly important effector cytokine	Eisenberg and Osterman, 1977; Eisenberg et al., 1980; Jerrells and Osterman, 1983; Kodama et al., 1987

## Effectors of oriential killing

Early *in vitro* experiments with macrophages showed that these cells could kill *O. tsutsugamushi* when activated by cytokines present in supernatants of activated splenocytes (Nacy and Meltzer, 1979; Nacy et al., 1983). Indeed, peritoneal macrophages from mice infected with *O. tsutsugamushi* (Gilliam strain) 9 days earlier were considerably more resistant to *in vitro* infection due, most likely, to their activated status (Nacy and Osterman, 1979). Furthermore, when immune serum was present in the assay, there was enhanced uptake and killing of *Orientia*, indicating that Fc receptor-mediated uptake of opsonized bacteria may be an important mechanism of oriential clearance. Also, non-specific activation of intraperitoneal macrophages confers protection to an otherwise lethal inoculum (Nacy and Meltzer, 1984). Notably, however, *in vitro* Karp strain infection of peritoneal macrophages from C57BL/6 mice did not result in significant TNF-α production while it did when they were infected with *Rickettsia conorii* (Jerrells and Geng, 1994). On the other hand, when TNF-α is provided exogenously, macrophages acquire the ability to inhibit the growth of *O. tsutsugamushi in vitro* (Geng and Jerrells, 1994). In contrast with the previous data, there is evidence that activated macrophages can mediate, at least in part, transient immunosuppression that is observed up to 1 month after subcutaneous challenge of BALB/c mice with Karp strain, which is, as explained earlier, a chronic infection model (Jerrells, 1985). In these mice, lymphocytes had a decreased proliferative response to concanavalin A stimulation, to an unrelated model antigen and to alloantigens in a mixed lymphocyte reaction, and those defects could be corrected by depleting adherent cells (macrophages); furthermore, macrophages from infected mice (before day 28 post-infection) could inhibit the response of normal splenocytes to mitogens. This effect could be inhibited with indomethacin; thus, prostaglandins were likely playing a role. More recently, a global transcriptome analysis of human monocytes infected *in vitro* with *O. tsutsugamushi* for 8 h in comparison with uninfected monocytes was reported (Tantibhedhyangkul et al., 2011). Thousands of genes participating in many cellular processes changed their expression. The authors highlighted the type I IFN response and the expression of an M1 program (a pro-inflammatory phenotype) because of their importance in the regulation of the immune response. Furthermore, many of the genes that changed expression significantly were also identified in a transcriptome analysis of peripheral blood mononuclear cells from four patients with confirmed scrub typhus. All these studies with monocytes and macrophages are relevant since these cells are not only effectors of immunity, but also secondary targets of oriential infection in humans (Moron et al., 2001). Knowledge of the responses of the primary early target (dendritic cells) and the major disseminated target (endothelial cells) would expand understanding of the innate and adaptive immunity to *O. tsutsugamushi*.

## Antigens and the pursuit of subunit vaccines

Characterization of antigens from *O. tsutsugamushi* was first approached with SDS-PAGE separation of lysates of purified bacteria (or membrane fractions) followed by elution from individual gel slices and ELISA with the eluate and sera from immune mice (Eisemann and Osterman, 1981). They identified six major immunoreactive fractions on the membranes and showed antigens that were common to three strains, but also some antigens that were recognized exclusively by homologous immune sera. A subsequent study using immunoprecipitation of radiolabeled proteins of *Orientia* showed that the most abundant proteins were also the ones recognized by immune sera (Hanson, 1985). Today, using Western blot, we know that there are five proteins of *O. tsutsugamushi* that are clearly immunodominant to humans. They have molecular weights of 22 kDa, 47 kDa, 56 kDa, 58 kDa, and 110 kDa (Chen et al., 2011). It is also important to point out that orientiae do not have peptidoglycan or LPS in their cell wall (Amano et al., 1987), which in other bacteria provide cross-reactive and protective antigens.

The first step toward the production of a subunit vaccine for scrub typhus was accomplished with the cloning and expression of two major immunoreactive proteins from the Karp strain of *O. tsutsugamushi*, the 110 kDa and 56 kDa oriential proteins, which are cross-reactive with homologous antigens in two other stains, Kato and Gilliam (Oaks et al., 1987; Stover et al., 1990a,b). Cloning the 56 kDa protein was very important because all convalescent sera from humans that had suffered scrub typhus have antibodies against this protein as determined by Western blot. Nevertheless, strain-specific epitopes were identified using monoclonal antibodies (Stover et al., 1990a) and deletion constructs of this gene (Choi et al., 1999); indeed, evidence from the amino acid sequences of multiple strains indicated that the 56 kDa protein has four major variable domains that result in different alleles for each strain (Ohashi et al., 1992). Antigens of 11 kDa and 58 kDa were cloned shortly thereafter and shown to be heat shock proteins recognized by the sera of humans who previously had scrub typhus (Stover et al., 1990b). The 58 kDa protein was considered of major interest because of its potential high-degree of conservation among different strains of *O. tsutsugamushi*. In 1991, the cloning of a 22 kDa protein from the Karp strain was accomplished; this protein was recognized by both antibodies and a CD4^+^ T cell line generated from mice immune to Karp strain (Hickman et al., 1991). This achievement was followed by the cloning of a 47 kDa antigen and the recognition of its ability to stimulate a similar anti-*O. tsutsugamushi* mouse CD4^+^ T cell line (Hickman et al., 1993). This study also reported a 20 amino acid peptide from this protein that specifically stimulated this T cell line.

Polyclonal hyperimmune serum from rabbits infected with *O. tsutsugamushi* inhibits entry of a homologous strain into target cells *in vitro* without dependence on complement activity (Hanson, 1983). Related subsequent investigations showed that antibodies produced in mice and rabbits against the recombinant 56 kDa protein from the Boryong strain inhibit attachment and penetration of mouse fibroblasts (Seong et al., 1997a). This finding prompted the authors to test it as a vaccine, and they found that i.p., immunization of C3H/HeDub mice with this recombinant protein, with alum as adjuvant, provided protection against otherwise lethal inocula of the homologous strain (Seong et al., 1997b). Part of the response was likely mediated by T cells as indicated by antigen-specific proliferation of lymphocytes from immune animals; these cells produced IL-2 and IFN-γ. High titer antibodies were also produced. In addition, another study demonstrated that certain monoclonal antibodies against the 56 kDa protein neutralized infection by a homologous *O. tsutsugamushi* strain inoculated i.p. (Seong et al., 2000). In cynomolgus monkeys, a truncated recombinant fragment from the 56 kDa protein of Karp strain, adjuvanted with Montanide ISA 51 and CpG 10103, stimulated production of appropriate antibody and T cell responses; however, it did not prevent the development of rickettsemia upon challenge 1 month after vaccination. In contrast, prior infection yielded complete immunity (Chattopadhyay et al., 2005). The 56 kDa antigen was also studied in the form of a DNA vaccine, which resulted in 60% survival of the challenged outbred mice after four intramuscular injections of the DNA (Ni et al., 2005). When the 56 kDa protein was expressed as a fusion protein with the 47 kDa antigen and used for immunization (in a Karp strain model), survival was better than that produced by either one of the two recombinant proteins (Yu et al., 2005), suggesting that a multisubunit vaccine could be a potentially successful approach. However, in this case, a whole cell lysate of Karp strain provided the best protection.

## Modern vaccinology concepts applied to scrub typhus

We recently reviewed some general principles of immunity and vaccine development, as it applies to *Rickettsia* (Valbuena, 2012); thus, we will mainly emphasize those that are particularly pertinent to *Orientia* (Table 4). The earliest attempts to develop a vaccine against scrub typhus have not been successful because they have not directly addressed two important characteristics of this infection, namely the significant antigenic variation among strains of *O. tsutsugamushi* and the limited cross-protective immunity that natural infections trigger against heterologous strains. The most recent approaches to vaccine development have focused on antigens that are immunodominant for the humoral immune response, and those attempts have used the previous paradigm of vaccinology, the trial-and-error approach. We believe that those studies have met with very limited success because they failed to consider the fact that scrub typhus is an intracellular infection for which cellular immunity is critical. Those studies also overlooked the fact that oriential immunodominant antigens do not trigger sterilizing protective immunity in a still unknown proportion of humans who develop a chronic infection with *O. tsutsugamushi*. Furthermore, there are data supporting the concept that antibodies, CD4^+^T cells, and CD8^+^T cells target different antigens (Moutaftsi et al., 2007). Thus, antigen identification through antibodies does not necessarily detect antigens recognized by CD4^+^T cells or, particularly, CD8^+^T cells.

**Table 4:** **Important immunological considerations for the design of a vaccine against scrub typhus**.

*Orientia tsutsugamushi*, the etiologic agent of scrub typhus, presents remarkable genetic heterogeneity among strains and particularly antigenic diversity of the major surface proteins *O. tsutsugamushi* can establish asymptomatic chronic infections in a still unknown portion of patients The number of strains of *O. tsutsugamushi* is large, and the naturally dominant immune response appears to be directed against poorly conserved or highly variable antigens Both antibodies and T cells appear to play important roles; however, homologous immunity wanes over time, and heterologous immunity is very short-lived There is very limited knowledge about the correlates of protective immunity against *Orientia* Endothelial cells, the main targets of the systemic oriential infection, are important physiological regulators of the immune response The identification of immunoregulators in the saliva of the vectors, larval trombiculid mites, has not been pursued

Advance toward development of a clinically effective vaccine against scrub typhus is currently hampered by many barriers; among them are the following: (1) the lack of comprehensive knowledge of antigens that are common to different *O. tsutsugamushi* strains and that, as a whole, can stimulate broad branches of the adaptive immunity (both cellular and humoral); (2) the limited knowledge about the signature markers (i.e., correlates of protection) of cross-protective immunity, an aspect that is complicated by the chronicity of the infection; and (3) the lack of appropriate small animal models for vaccine testing that recapitulate the systemic endothelial infection observed in humans and the percutaneous transmission by chiggers.

Current mouse models of scrub typhus using intraperitoneal inoculation have provided some information about the anti-oriential immune response; however, those models do not produce systemic endothelial infection, which is the hallmark of human infections with *O. tsutsugamushi*. Therefore, they cannot address how *O. tsutsugamushi* is cleared from endothelial cells because normally secondary targets of infection, macrophages and mesothelial cells, are the main targets of infection in mice infected intraperitoneally. Discovery of mechanisms of protective immunity and identification of correlates of protective immunity are only possible in the context of an animal model in which endothelial cells are the main target cells of infection because successful effector mechanisms need to be active in or against endothelial cells. This consideration is important because endothelial cells interact closely with leukocytes as part of their physiological function. Some of these aspects as related to rickettsioses have recently been reviewed (Valbuena and Walker, 2006, 2009).

We propose that a modern approach to vaccine development against scrub typhus will require the development of more appropriate animal models and should be guided by two general overarching principles: (1) identification of relevant antigens that, in combination, can stimulate all relevant branches of adaptive immunity, namely antibodies, CD4^+^ T cells, and CD8^+^ T cells; and (2) definition of immunological correlates of protection to guide the selection of vehicles, vectors, schedules, and adjuvants. In this regard, it will be important to identify new correlates of protection and/or adapt modern correlates of protection identified in humans and other models of intracellular infection. For instance, now we know that there is functional heterogeneity of the T cell effector responses (including cytokine secretion, cytolytic activity, and development of various memory phenotypes) and that there are particular subsets of T cells, which express unique combinations of effector functions, that are more protective (Wu et al., 2002; Darrah et al., 2007; Miller et al., 2008; Akondy et al., 2009; Lindenstrøm et al., 2009).

Given that human adaptive immunity against *O. tsutsugamushi* (as well as that of NHPs) is not sufficiently cross-reactive, wanes over time, and is not even sterilizing, it will be difficult to identify truly predictive correlates of protective immunity from the analysis of human or NHP samples. A possible solution to identifying relevant correlates of protective immunity might be found in the analysis of immunity against spotted fever rickettsioses (*Rickettsia* is phylogenetically the closest to *Orientia*) since immunity against the rickettsiae that cause spotted fevers is sterilizing and long lasting (Valbuena, 2012); however, one problem is that there is still very little information about the T cell response of humans against *Rickettsia*. An alternative source of information about correlates of protective immunity against intracellular pathogens in humans can be derived from the study of the immune response against the two most successful vaccines (that include T cell immunity) produced thus far, namely vaccinia, the smallpox vaccine, and 17D, the yellow fever vaccine (Gaucher et al., 2008; Akondy et al., 2009; Querec et al., 2009). Excellent reviews on these topics have been published recently (Miller et al., 2008; Pulendran, 2009; Liu et al., 2010; Ahmed and Akondy, 2011). The most important points to highlight are that effective immunity can be predicted by the integrated analysis of multiple parameters including the following: (1) the rapidity and magnitude of the primary T cell response (as determined by increased expression of activation markers such as HLA-DR, CD38, Ki-67, CD154, and decreased expression of Bcl-2); (2) effector CD8^+^ T cells expression of IFN-γ, perforin and granzyme B; (3) effector and memory cells production of a mixture of various cytokines (even mixed Th1/Th2 cytokines); (4) memory cells that have a bimodal distribution consisting of both effector memory and central memory phenotypes; (5) rapid activation of both innate (especially NK cells) and adaptive immune cells; (6) persistence of the response over time; (7) early up-regulation of master transcriptional regulators of innate and adaptive immunity such as ETS2, IRF1, IRF7, IRF8, STAT1, and FOXO3a.

Our current knowledge of immunity against *O. tsutsugamushi* from animal models and natural and experimental human infections suggests the following features: (1) humoral and cellular adaptive immunity act in concert to produce the best outcomes; (2) protective immunological memory against heterologous strains is very short-lived; and (3) the protective memory response against homologous strains lasts much longer but also seems to wane over time. The following scenario could explain these phenomena: during natural *O. tsutsugamushi* infections, immunodominant antigens recognized by the humoral and cellular immune responses are mainly encoded by genes that are not conserved among different strains of *O. tsutsugamushi*, while conserved genes encode subdominant or cryptic antigens. Since the response against immunodominant antigens results in a “suppressed” or masked response against subdominant antigens, the resultant absolute number of possible memory cells capable of recognizing conserved (i.e., cross-reactive) antigens is very limited and is expected to wane more rapidly. There are compatible data in support of this scenario for the anti-oriential humoral immune response since immunodominant antigens, such as the 56 kDa surface proteins, are highly variable antigens among different strains of *O. tsutsugamushi*, and these antigens provide very limited cross-protection.

We believe that immunization with a combination of conserved *Orientia* antigens recognized, as a whole, by CD4^+^ T cells, CD8^+^ T cells, and antibodies will lead to anti-oriential immunity that is sterilizing, cross-reactive, and sustained. For the identification of the relevant antigens, we advocate the use of an empirical approach to antigen discovery, i.e., testing of all possible conserved antigens for immunogenicity (for both T cells and B cells) and protection because there is not sufficient evidence that predictions from the bioinformatics approaches of reverse vaccinology (analysis of complete microbial genomes to predict immunogenic proteins based on predefined rules derived from the analysis of large empirical datasets) are truly comprehensive and maximally accurate. Although these approaches have generally identified protective antigens (Rinaudo et al., 2009; Sette and Rappuoli, 2010), an entire microbial genome has not been experimentally screened in order to determine the proportion of truly antigenic proteins that were predicted through bioinformatics approaches. There is experimental evidence that those predictions are very limiting (Wu et al., 2011). In fact, using predictive algorithms in bioinformatics approaches, known protective bacterial antigens actually have less predicted epitopes recognized by T cells than randomly selected bacterial protein sets used as a control (Halling-Brown et al., 2008).

**Figure 1:**
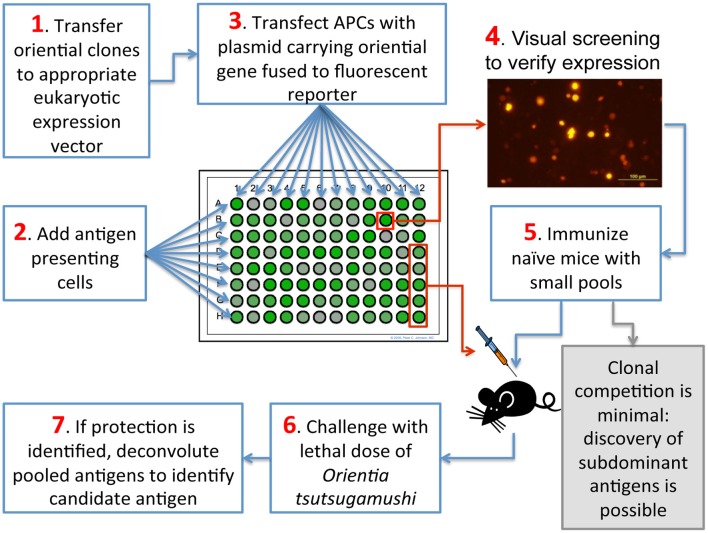
**Diagram of a strategy to discover subdominant antigens recognized by T cells**.

A comprehensive discovery program for cross-reacting antigens of *O. tsutsugamushi* guided by genomic information, which has not been attempted to date, is likely to yield the most relevant vaccine candidates. This approach is now possible because genomic information, although limited to two strains, is now available (Cho et al., 2007; Nakayama et al., 2008, 2010) and is expected to keep growing. The construction of libraries with all conserved open-reading-frames (ORFs) of *Orientia* (the ORFeome) is possible through PCR-mediated amplification of those ORFs or through gene synthesis; those genes can then be expressed in histocompatible antigen presenting cells (APCs). Such a project is feasible because the number of conserved genes is not excessive; if one includes the genomes of several species of *Rickettsia* to determine the most conserved genes, the number is about 500 genes (Nakayama et al., 2010). Although anti-*Orientia* immune T cells could be used for screening and antigen detection, those T cells would necessarily be memory cells that can only recognize immunodominant epitopes; that is, of course, the nature of a physiological immune response. Thus, the resultant candidates may not be protective because, as explained previously, *O. tsutsugamushi* can establish chronic infections in the face of an immune response against immunodominant antigens. Consequently, we propose that a better antigen identification method would consist of *in vivo* screening in immunologically naive animals (Figure 1); such animals would be vaccinated with APCs expressing conserved ORFs from *O. tsutsugamushi* or, alternatively, with appropriate vaccine DNA vectors expressing those ORFs. This second option is similar to expression library immunization (ELI) (Sykes, 2008). This method, like the one we propose, allows the priming of naïve T cells by the expressed cloned microbial genes regardless of whether they are subdominant or dominant during a natural infection as long as the appropriate T cell receptors are present; however, it relies on a DNA immunization strategy so antigen expression is not guaranteed in all cases. In either method, protection from challenge with *O. tsutsugamushi* would identify subdominant or cryptic antigens among those proteins of *O. tsutsugamushi* encoded by conserved genes. This approach is likely to identify immunogenic and protective proteins from the normally subdominant pool of proteins. This outcome will likely be the case given the evidence from other models of intracellular infection that vaccination against subdominant epitopes can produce effective cellular immune responses (Riedl et al., 2009; Ruckwardt et al., 2010; Im et al., 2011). An additional consideration that would support our approach is that, from the perspective of the T cell response, cross-reactivity is also favored by the T cells themselves since the T cell receptor is multispecific due to structural flexibility and interactions with only a few residues within the presented peptides. Thus, the T cell repertoire has a tremendous capacity for cross-reactivity (Mason, 1998; Sewell, 2012). If resources are not available for testing all conserved ORFs, the testing could be prioritized using other criteria including antigenicity predictions (with the caveats already mentioned) and levels of expression of bacterial proteins *in vivo* (Rollenhagen et al., 2004; Mahdi et al., 2012).

Unlike the system just proposed, most other techniques for identification of antigens recognized by T cells use immune T cells (i.e., memory T cells) to detect positive reactions; therefore, they are biased by immunodominance, which is, as mentioned earlier, not useful in the case of scrub typhus. Examples of those techniques include screening of retrovirus-based cDNA libraries (Wang et al., 1998; Aoshi et al., 2005), use of random expression genomic libraries in *E. coli* to feed macrophages as APCs (Gonçalves et al., 2006), screening of purified proteins (from a genomic library) coupled to beads (Valentino et al., 2011a,b), screening of synthetic minigene libraries (Hondowicz et al., 2012), and screening of plasmid-encoded combinatorial peptide libraries (Siewert et al., 2012).

**Figure 2:**
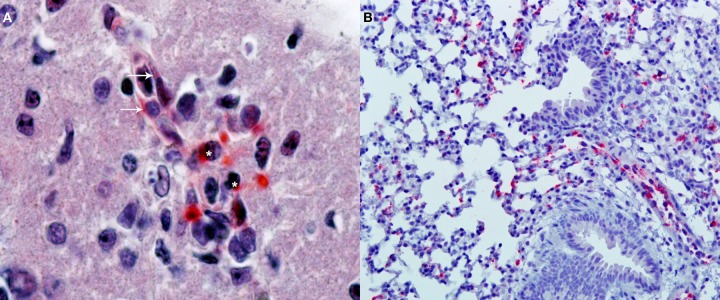
**Immunohistochemical detection of oriential antigen. (A)** Analysis performed in the brain of a C3H/HeN mouse infected i.v., with 2 × 10^5^ FFU of *Orientia tsutsugamushi* 11 days earlier (original magnification 400×). Alkaline phosphatase signal from the secondary antibody produces a red precipitate visible in endothelial cells of a microvessel (arrows) and in adjacent mononuclear leukocytes (asterisks). The section is counterstained with hematoxylin and eosin. **(B)** Analysis of the lungs of the same animals (original magnification 100×) with the same alkaline phosphatase technique shows multiple foci of oriential antigen in the alveolar walls. Courtesy of Thomas Shelite.

After the identification of a combination of antigens that together can stimulate protective responses mediated by CD8^+^T cells, CD4^+^T, and B cells (antibodies), the next developmental step for a modern anti-*Orientia* vaccine will be the selection of the best strategy to optimally stimulate those immune system cells. The objective is to do better than nature by testing different adjuvants, immunization routes, and schedules. These concepts are evolving very rapidly; thus, the reader is referred to recent reviews (Coffman et al., 2010; Liu, 2010). This testing must be done in appropriate animal models, thus the generation of relevant animal models to identify correlates of protective immunity and to test vaccine candidates is essential in the vaccine development effort against scrub typhus. Non-human primates constitute excellent models because scrub typhus pathogenesis and immunity are very similar to that of humans; however, the high cost of these animals together with the necessity of performing experiments with *O. tsutsugamushi* in a biosafety-level-3 (BSL-3) environment preclude their general and consistent use. Although there are fundamental biological species differences between humans and mice that are likely to affect direct translation to humans (Marian, 2011), very valuable information can be obtained from mice as long as there is a serious attempt to mimic the pathophysiology of humans. The ideal model should include transmission by chiggers, and some progress has already been made in that direction (see earlier sections on this topic). Alternatively, intradermal injection of *O. tsutsugamushi* to mimic the natural path of infection may be more reproducible and easier to implement. Our group is currently developing this model in C3H/HeN mice infected with the Karp strain; results are very promising since the infection is systemic and predominantly endothelial (Figure 2). We have also developed an intravenous infection model with similar results; the reports of these models are forthcoming. Another model that may provide an improved bridge to direct translation for human studies is that provided by humanized mice. These are highly immunodeficient mice (Hiramatsu et al., 2003; Brehm et al., 2010; McDermott et al., 2010) transplanted with human hematopoietic stem cells to reconstitute the human hematolymphoid system. Since these humanized mice can mount adaptive human immune responses, they provide a relevant model for translational research (Shultz et al., 2007) and the study of infectious diseases (Rämer et al., 2011).

## Conclusion

Scrub typhus is a truly neglected vector-transmitted intracellular bacterial infection that causes significant morbidity and mortality in a large geographical area of the world. A prophylactic vaccine is a public health priority because of its high incidence, high mortality, non-specific clinical presentation, lack of sensitive diagnostic tests, and emergence of antibiotic resistance. For the last 60 years, continuous efforts to develop a vaccine have not succeeded because they have not been able to address and overcome the inability of the natural immune response against *Orientia* to produce sterilizing, long-lasting, and cross-protective immunity. This inappropriate immune response appears to be a consequence of natural immunodominance of antigens encoded by oriential genes that are not conserved among a large number of strains. However, current technological advances, together with approaches rooted in a deep general understanding of effective human immune responses, will help address the current barriers to vaccine development against *Orientia tsutsugamushi*.

### Conflict of interest statement

The authors declare that the research was conducted in the absence of any commercial or financial relationships that could be construed as a potential conflict of interest.
